# Electric vehicle charging stations in the workplace with high-resolution data from casual and habitual users

**DOI:** 10.1038/s41597-021-00956-1

**Published:** 2021-07-07

**Authors:** Omar Isaac Asensio, M. Cade Lawson, Camila Z. Apablaza

**Affiliations:** 1 School of Public Policy, Georgia Institute of Technology, Atlanta, GA USA; 2 Institute for Data Engineering & Science, Georgia Institute of Technology, Atlanta, GA USA; 3 School of Economics, Georgia Institute of Technology, Atlanta, GA USA

**Keywords:** Energy infrastructure, Environmental economics, Energy and society, Business and industry

## Abstract

Problems of poor network interoperability in electric vehicle (EV) infrastructure, where data about real-time usage or consumption is not easily shared across service providers, has plagued the widespread analysis of energy used for transportation. In this article, we present a high-resolution dataset of real-time EV charging transactions resolved to the nearest second over a one-year period at a multi-site corporate campus. This includes 105 charging stations across 25 different facilities operated by a single firm in the U.S. Department of Energy Workplace Charging Challenge. The high-resolution data has 3,395 real-time transactions and 85 users with both paid and free sessions. The data has been expanded for re-use such as identifying charging behaviour and segmenting user groups by frequency of usage, stage of adoption, and employee type. Potential applications include but are not limited to simulating and parameterizing energy demand models; investigating flexible charge scheduling and optimal power flow problems; characterizing transportation emissions and electric mobility patterns at high temporal resolution; and evaluating characteristics of early adopters and lead user innovation.

## Abstract (summary)

**Table:** 

Measurement(s)	Unit of Electric Charge
Technology Type(s)	Internet Mobile Technology
Factor Type(s)	charging station location
Sample Characteristic - Environment	office
Sample Characteristic - Location	contiguous United States of America

Machine-accessible metadata file describing the reported data: 10.6084/m9.figshare.14357123

## Background & Summary

A combination of range improvements, falling prices, and accelerated investments in charging infrastructure have projected the EV industry to capture an increasing share of the automotive market from conventional internal combustion engine vehicles. By 2040, analysts estimate that EVs will make up 57% of all passenger vehicle sales and over 30% of global passenger vehicle fleets^1^. Given the digital transformations in energy and transportation over the coming decades, there will be an influx of data and activity about human mobility and electrified transport that will benefit from grid-connectivity and real-time analysis. However, today there are several practical challenges that limit the ability of researchers and policymakers to access high-resolution data about electricity use in emerging transportation infrastructure. For example, station hosts may collect or monetize data about their customers’ usage patterns and other trends to manage station use. This decentralized decision-making is especially important during peak times as station hosts set price policies. However, station hosts currently have little to no incentive to share data or information with other stakeholders. This limited real-time data availability is largely due to the absence of regulation that would allow for: (i) easy data sharing across regions or utility jurisdictions, (ii) incentives for network interoperability, (iii) widespread technology standards, and (iv) accessible proprietary digital platform data with built-in privacy protections or privacy preserving controls for independent analysis.

The rapid deployment of EVs has generated many new potentially informative data streams related to user schedules and preferences, charging profiles and power system requirements. However, because most charging stations are typically owned by private entities in highly decentralized models of station growth, real-time EV data is concentrated by digital platforms or network providers. This has led to high costs for data aggregation and record linkage. As a result, the high-resolution data that is needed to inform smart grid or transportation policies has remained hard to access, leaving researchers and analysts with few alternatives to expensive and infrequent government transportation surveys. In addition, most EV charging stations are surprisingly not independently sub-metered and suffer from poor network interoperability^2^. This has made it difficult to investigate patterns of charging demand at the high resolution needed to understand individual-level behaviour. For example, high temporal resolution data is needed to evaluate the effectiveness of real-time or dynamic pricing policies^3^, which are critical for large-scale system optimization and demand and capacity planning. This situation has created practical challenges for owners, firms, utilities, regulators, and others in making effective planning decisions for infrastructure investment or performance evaluation. Consequently, prevailing methods have primarily relied on the use of simulated data^4^, self-reported surveys^5,6^ or interview data^7^, which do not contain real-time information and sometimes present challenges such as recency or self-report bias.

In this paper, we describe a high-resolution dataset of EV charging transactions from a large workplace charging program that is resolved at the station level with sub-metering capabilities. This includes real-time data about charging usage from various EV consumer types such as managers and non-managers, casual and habitual users, and early and late adopters in a large corporate setting. This type of information could be of use to EV station hosts, consumers, utilities, regulators, researchers, and others who have a need to understand the impacts of EV station consumption at a micro-level. The data also provides critical information needed to optimize revenue models and resource utilization strategies, especially during periods of rapid user growth. At a research level, this data could be used by scholars to address questions in several possible areas of inquiry. These include, but are not limited to, energy modelling and load profiling^8–11^, power systems optimization^12–16^, economic and policy analysis^17–20^, energy data analytics and visualization^21–23^, management and innovation studies^24–26^, consumer behaviour research^9,27–29^, and life cycle analyses^30,31^.

## Methods

The database contains high-resolution data for 3,395 electric vehicle charging sessions from one of the largest workplace charging programs in the United States. Each charging session contains rich information about the user who logged the session (such as vehicle type, commute distance, and manager or non-manager status), unique identifiers for the specific charging station and the location where a session was initiated, as well as various usage characteristics (energy output in kWh, time spent charging, and U.S. dollars spent). All sessions were recorded under a set of conditions that were common to all multi-site charging locations, as shown in Table 1. For example, many of the factors that influence charging behaviour have been standardized, including site characteristics of the workplace, station characteristics, and the pricing model. In the next section, we discuss the specific data collection and experimental features implemented across the firm’s charging stations.

**Table 1:** Experimental features.

*Site features*	
Charger site	Large workplace
Workplace type	Manufacturer
Geographic region	Midwest United States
*Station characteristics*	
Charger model	GE WattStation
Charger type	Level II
Station host	Employer
Access	Restricted to employees and visitors
Plug type	SAE J1772 standard
*Pricing model*	
Payment platform	Standard and mandatory for all transactions
Pricing type	Time-based
Revenue scheme	Dynamic; free for 4 hours, then $1/hr.
Parking cost	Free

### Digital data collection

All data was collected and aggregated automatically on a mobile platform used by employees at the firm. Users could view their EV charging time and visualize their session-level energy consumption and any payments to a network operator. Each of the firm’s charging stations has the capability to report real-time statistics about the energy use measured to the nearest hundredth of a kilowatt-hour and the duration of a session measured to the nearest second. In addition, the payment platform automatically reports the cost of charging transactions to the nearest cent based on direct kWh measurement. All data collection was digital and did not require human readings. This lack of potential for human error normally associated with manual data entry and validation only enhances the reliability of the real-time information presented within the dataset.

Charging stations were installed at a rate of approximately 2 stations per week over the course of a year. In this way, the collected data provides a window into the growth of the program over time and into the specific behaviour exhibited by users of different types (see Breadth and Coverage). Because data is pulled from stations at multiple facilities operated by the firm, it is possible to understand heterogeneity in program use and load profiles at different types of facilities. In separate analyses, we confirmed that the installation of new stations did not cannibalize the charging use of the existing stations.

### Price scheme and experimental structure

To verify the pricing scheme and design features for this dataset, we conducted a series of interviews with the internal operators of the firm’s EV charging program. We collected information about the motivations guiding the program implementation and the specific costs faced by EV users. Here, we summarize key details about the price policy, usage rules, and communications between the program operators and EV users.

Because subsidized electric vehicle charging is intended to be a workplace perk for the firm’s employees and visitors, the price policy was set up to allow for 4 hours of free (Level 2) charging. After this point, a small fee of $1 per hour (including a $0.50 transaction fee) was assessed. This nominal fee reflected the firm’s desire to mitigate congestion and promote the accessibility of its charging stations for a significant share of daily commuting needs. The price policy was not intended to maximize revenue or quickly recoup capital costs. It is fairly typical of price policies at large workplace charging programs in the U.S.

The charging stations were accessible to registered users 24 hours per day. Access to the stations was restricted to employees and visitors only. Because payment was processed through the mobile platform, users were also required to register before using a charging station. Next, we describe the communication between program operators and users.

Employees received periodic website and email communications regarding EV access rules and policies. Employees at high-demand facilities were also sent reminders about resource sharing. This behavior by program hosts to include behavioral messaging can be considered a leading indicator of strategies for managed infrastructure in high growth and space-constrained environments^3^. In the next section, we share our protocols for pre-processing and anonymizing the data.

### Pre-processing and anonymization

Because of the highly granular and automated nature of the data collection, only light pre-processing was conducted prior to publishing the dataset for reuse. A small number of transactions were removed for noncompliance with the price scheme because they either erroneously failed to charge for a session lasting more than 4 hours or charged a user for a session lasting fewer than 4 hours. In addition, any observations that contained missing fields for session cost and kWh used were removed because they indicated errors in data reporting or with the charger’s hardware. We confirmed that these data cleaning procedures did not statistically change the means (t-test p-value > 0.10) or the distribution (K-S test D-statistic 0.0023) of kWh usage in the final dataset. Some additional ancillary fields were also removed because they contained no new information. This includes a variable for whether a session failed due to technical error; and a network variable which was common to all sessions.

The dataset does not include any personally identifiable financial or banking information. Due to privacy restrictions, we carefully anonymized the dataset before publication to remove any potential identifying features. For example, we removed the home zip codes of users, the zip codes of the charging stations, and the make and model of vehicles. To mitigate the possibility of reidentification, all unique identifiers of people or locations, including user, station, and location identification numbers, were pseudonymized before publication.

Date and time fields were converted from AM/PM format to a more machine-friendly 24-hour format. Next, we discuss attributes of our derived variables.

### Derived variables

To assist with further analyses, we include several derived variables from the raw data in Table 2. Because many transportation studies focus on trip distances, we calculated an estimate of commute length by finding the distance from the centroid of a user’s self-reported home zip code to the address of a station. This distance is estimated in miles and is only available in cases where a user decided to add a home zip code to its mobile platform profile. Although not all users reported a home zip code, the raw data included full addresses for all charging stations. In addition, we include dummy variables to identify the specific days of week that a charging session took place. We also provide a variable for the number of total sessions logged by a user, calculated by grouping transactions by unique user ID, and a variable indicating whether the user provided a zip code. The variable containing facility type was recoded from free text to a numerical indicator for where a charger is located: at either a manufacturing facility, office facility, research and development facility, or other facility type.

**Table 2:** Data dictionary.

Variable	*Description*
*Unique identifiers*	
Session	Identifies a specific EV charging session, where each row in the dataset represents a single session.
User	Identifies a specific electric vehicle owner. A user who charges multiple times can be identified throughout the dataset using this field.
Station	Identifies a specific EV charging station which indicates where a given charging session occurred.
Location	Identifies a given building or location, operated by the firm, where one or more EV chargers is available.
*Session characteristics*	
Create stamp	The timestamp at which a charging session was initialized, in YYYY-MM-DD HH:MM:SS format.
End stamp	The timestamp at which a charging session was terminated, in YYYY-MM-DD HH:MM:SS format.
Charge time	The duration of a charging session measured in hours.
Cost	The amount charged for the charging session in dollars, per the price policy implemented by the firm.
Total kWh	The total energy use for a given charging session, measured to the nearest hundredth of a kilowatt-hour.
Days of week	Binary variables indicating the specific day of week on which a given transaction was logged.
Facility type	Maps a given transaction to the type of facility where it took place. Manufacturing facilities correspond to 1, office facilities to 2, research and development to 3, and other to 4.
*User characteristics*	
Manager vehicle	A binary variable indicating whether the vehicle associated with a given charging session is of the type generally owned by the firm’s managers as a result of a corporate incentive program (1 if manager vehicle, 0 if not).
Early adopter	A binary variable indicating whether a given user was an early adopter or late adopter of the EV charging program (1 if early adopter, 0 if late adopter). Early adopters are defined as the first quartile of users to log a charging session, while late adopters are defined as the remaining users.
Habitual user	A binary variable indicating whether a user is a casual or habitual user of workplace charging (1 if habitual, 0 if casual). A habitual user is defined as someone who logged more than the median of 19 charging sessions over the course of the data collection period, while a casual user is someone who logged fewer than 19 sessions.
Reported zip	A binary variable indicating whether a user self-reported a zip code to the network operator.
Platform	The type of device used to register a session. One of Android, iOS, or Web.
Distance	The estimated distance in miles from the centroid of a user’s provided zip code to the exact position where the charging station is located. Not all users provided a zip code.
Total sessions	The count of total sessions logged by a given user over the course of the observation period.

For convenience, we also created three new variables to identify different user types. The variable indicating casual and habitual users was derived by observing the number of repeat transactions per user during the study. Next, the variable indicating early and late adopters was derived by looking at the date of each user’s first transaction. Finally, the binary variable indicating whether a vehicle is of the type commonly used by managers was derived from a variable which initially listed the specific make and model of the vehicle logging a specific charging session. We were able to identify a subset of users in the dataset who drive a vehicle commonly used by the firm’s management-level staff due to a corporate incentive program. We provide this information in the dataset.

### Ethical approval

Protocols for secondary data processing were reviewed by the Georgia Tech Institutional Review Board (IRB) under approved protocol H19304. Users provided informed consent to have their personal data collected, used, transferred to and processed in the United States. No personally identifiable information (PII) was collected or shared.

## Data Records

All data relevant to this publication is stored in a single machine-readable, comma-separated value file made available at the following address within Harvard Dataverse^32^: 10.7910/DVN/QF1PMO.

### Description of features

The data contains a total of 24 features describing various attributes of a charging session, user, or station. A detailed description of all fields available in the published dataset can be summarized in Table 2.

### Breadth and coverage

The breadth of the dataset includes complete observations from 25 different facilities and 105 deployed charging stations at a large workplace campus operated by the firm. The coverage of the dataset includes high-resolution transactions collected continually over a 24-hour cycle, including work and non-work hours spanning multiple shifts with valid but minimal overnight charging. A total of 85 registered users logged at least one session over the course of the data collection period, which ran for 46 weeks from November 2014 until October 2015. During this period, new stations were added at a rate of approximately 2 stations per week to the network. Next, we describe the distributions of charging transactions and users’ segmentation.

**Fig. 1:**
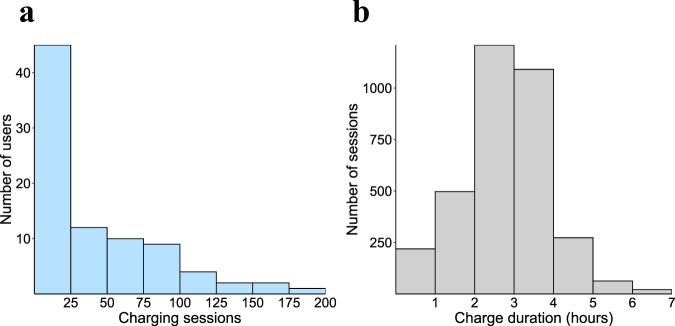
Histograms describing behaviour of users related to charging session duration and number of charging sessions. **(a)** Histogram displaying the frequency of charging sessions by users. **(b)** Histogram displaying the distribution of charging session length, measured in hours. A small number of outlier observations are not shown.

In Fig. 1a we show the distribution of total sessions per user, which ranges from 1 to 191 transactions. A significant number of users charged more times than the global average of 40 transactions per user. This indicates that many users relied on workplace chargers at least once per week to meet their daily commute needs. To investigate the sessions further, we then display the number of sessions by charging duration in Fig. 1b. It shows a normally distributed charging duration with a mean of 2.88 hours, and most transactions terminating within an 8-hour shift time. In the next section, we validate differences among user types in order to illustrate differences in behaviour between casual and habitual users, early and late adopters, and managers and non-managers. This is important because to date there has been very little evidence about user heterogeneity and its impact on EV charging consumption

We identify three ways of segmenting user types in the sample. Table 3 classifies transactions for user types according to their frequency of usage, stage of adoption of workplace charging infrastructure, and employee type. First, we distinguish between casual and habitual users by frequency of repeat usage. We define casual users as those who charged fewer than the median of 19 times and habitual users as those who charged 19 times or more. We find that casual users are statistically different in duration of charging session, kWh consumption, repeat transactions per user, and commute distance. However, we find that casual and habitual users are statistically similar in session revenue.

**Table 3:** Descriptive statistics according to user types.

Frequency of usage	Casual users (<median number of sessions)					Habitual users (>median number of sessions)					p-value
	Mean	SD	Min	Max	Total sessions	Mean	SD	Min	Max	Total sessions	
Duration of charging session (hours)	2.7	1.34	0.02	9.32	276	2.85	1.52	0.01	55.24	3119	0.08
Total consumption (kWh)	5.17	2.57	0	16.94	276	5.87	2.91	0	23.68	3119	0.00
Repeat transaction per user (count)	6.57	5.1	1	18	276	72.53	41.89	19	192	3119	0.00
Session revenue ($)	1.34	1.56	0.5	5.75	26	1.04	1.04	0.5	7.5	353	0.34
Estimated daily commute distance, one way (mi)	11.84	10.24	0.97	40.93	202	19.26	11.32	0.86	43.06	2188	0.00
**Stage of adoption**	**Early adopters (first quartile of users)**					**Late adopters (excludes first quartile of users)**					**p-value**
	**Mean**	**SD**	**Min**	**Max**	**Total sessions**	**Mean**	**SD**	**Min**	**Max**	**Total sessions**	
Duration of charging session (hours)	2.78	1.9	0.01	55.24	1331	2.88	1.19	0.01	11.59	2064	0.07
Total consumption (kWh)	5.44	3.22	0	23.68	1331	6.05	2.63	0	20.38	2064	0.00
Repeat transaction per user (count)	63.38	56.3	1	170	1331	32.25	37.48	1	192	2064	0.03
Session revenue ($)	1.04	0.9	0.5	5.75	150	1.08	1.19	0.5	7.5	229	0.71
Estimated daily commute distance, one way (mi)	12.71	8.19	2.5	32.06	703	21.16	11.66	0.86	43.06	1687	0.00
**Employee type**	**Managers**					**Non-managers**					**p-value**
	**Mean**	**SD**	**Min**	**Max**	**Total sessions**	**Mean**	**SD**	**Min**	**Max**	**Total sessions**	
Duration of charging session (hours)	2.96	1.67	0.01	55.24	2022	2.67	1.2	0.01	8.71	1373	0.00
Total consumption (kWh)	5.89	2.26	0	19.06	2022	5.69	3.63	0	23.68	1373	0.06
Repeat transaction per user (count)	42.12	43.28	1	192	2022	37.11	46.75	1	170	1373	0.61
Session revenue ($)	1.07	1.12	0.5	7.5	256	1.03	1.01	0.5	5.75	123	0.71
Estimated daily commute distance, one way (mi)	21.04	11.77	0.86	43.06	1453	14.87	9.73	1.47	40.93	937	0.00

Second, the data also allows for comparison of early adopters and late adopters by the stage at which they started using the charging stations. For this analysis, we define early adopters as the first quartile of users using the charging stations available and late adopters as the remaining quartiles^33^. Although there has been some debate whether lead users of EVs are representative of the population who adopt EVs at a later stage, we find that early adopters are very similar in expected session revenue to late adopters. We also find evidence of some key differences between early and late adopters. Late adopters in this population drive larger one-way daily commute distances to the workplace and, as a result, they also consume more kWh per session (see Table 3). Given these characteristics, it should be possible to study alternative incentives that allow for faster recovery of capital investments by station hosts. For capacity planning, the data does not show evidence of major differences in the duration of charging sessions between early and late adopters. In future work with this dataset, this could be further investigated to understand distributional impacts and scenarios for demand modelling.

Third, we distinguish the characteristics of charging behaviour of managers and non-managers. We find that managers are relatively higher users of the EV charging stations on average, considering duration of charging sessions, kWh consumption, and repeat transactions. Further work could study normative strategies in the workplace that could be deployed to address overconsumption by managers. See for example ref. ^3^.

## Technical Validation

To validate the data and its documentation, we conducted interviews with the program managers to understand the charging access rules, characteristics of the charging sites, and the objectives of the workplace charging program. Consequently, we were able to verify the cost for each charging session using the published pricing scheme for the firm’s network of charging stations. In addition, we were also able to inspect the empirical charging rates by plotting the kWh drawn over time. We verified that none of the transactions could exceed the theoretical maximum charging rate, thus lending additional verification to the veracity of the measured electricity transfer. We find a high degree of reliability with the ability to translate each transaction into time series data for further analysis.

**Fig. 2:**
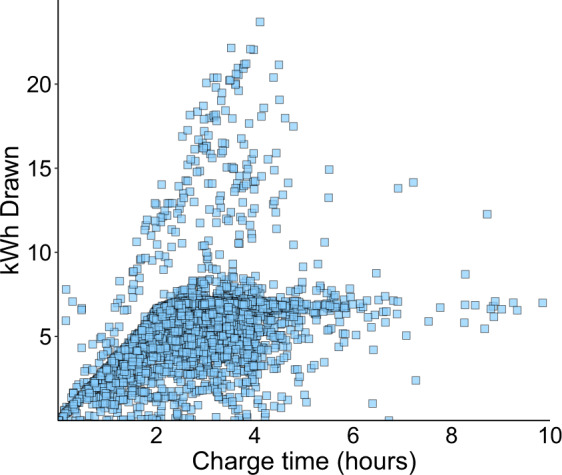
Plot of Level 2 charging service delivery.

We also validated that all transactions are within the range of charging rates for Level 2 charging stations, e.g. 3kWh – 19 kWh^34^. From Fig. 2, it is possible to identify two distinct charging rates as shown by the apparent slope of the curves in the charging service delivery profile. The lower curve follows the majority of the typical datapoints. These reflect car models whose charging rates range between 3.3 kW and 3.7 kW (Fig. 2). The steeper curve corresponds to car models with larger charging capacities. One limitation of this data is that as car make and model are self-reported, we do not have complete car battery info for all transactions. Machine or load disaggregation algorithms could be used to impute the missing data.

Regarding the generalizability of this data, the user behaviour reported here is typical of large workplace charging programs such as those documented in the U.S. Department of Energy’s Workplace Charging Challenge^35^. This was a DOE leadership initiative to encourage large corporate partners to begin to offer EaV charging services to employees. For example, through December 2016, there were already 400 participating firms, educational institutional and local governments who installed and planned to install an average of 18 stations per workplace program. By comparison, our sample of 105 networked stations represents one of the largest such programs. Additionally, other performance measures such as the timing of station plug-ins and plug-outs could be compared to other external workplace locations where similar user behaviour can be benchmarked and reasonably anticipated. It is also possible to benchmark the data with charging behaviour at other points of interest such as retailers, restaurants, or transit centres, see refs. ^3,20^.

The authors welcome questions, comments, or opportunities to collaborate with interested researchers.

## Data Availability

Replication code to support the findings of this study has been deposited to the following GitHub repository^36^: 10.5281/zenodo.4408528.
